# Advantages of Bayesian monitoring methods in deciding whether and when to stop a clinical trial: an example of a neonatal cooling trial

**DOI:** 10.1186/s13063-016-1480-4

**Published:** 2016-07-22

**Authors:** Claudia Pedroza, Jon E. Tyson, Abhik Das, Abbot Laptook, Edward F. Bell, Seetha Shankaran, for the Eunice Kennedy Shriver National Institute of Child Health and Human Development Neonatal Research Network

**Affiliations:** Center for Clinical Research and Evidence-Based Medicine, McGovern Medical School at The University of Texas Health Science Center at Houston, 6431 Fannin St, MSB 2.106, Houston, TX 77030 USA; Social, Statistical and Environmental Sciences Unit, RTI International, 6110 Executive Blvd., Suite 902, Rockville, MD 20852-3903 USA; Department of Pediatrics, Women & Infants Hospital of Rhode Island, The Warren Alpert Medical School of Brown University, 101 Dudley Street, Providence, RI 02905 USA; Department of Pediatrics, University of Iowa, 200 Hawkins Drive, Iowa City, IA 52240 USA; Department of Pediatrics, Neonatal-Perinatal Medicine, Wayne State University, Children’s Hospital of Michigan, 3901 Beaubien Blvd., 4H46, Detroit, MI 48201 USA

**Keywords:** Bayesian methods, Factorial trial, Hypothermia, Phase III trial, Stopping rules, Trial monitoring

## Abstract

**Background:**

Decisions to stop randomized trials are often based on traditional *P* value thresholds and are often unconvincing to clinicians. To familiarize clinical investigators with the application and advantages of Bayesian monitoring methods, we illustrate the steps of Bayesian interim analysis using a recent major trial that was stopped based on frequentist analysis of safety and futility.

**Methods:**

We conducted Bayesian reanalysis of a factorial trial in newborn infants with hypoxic-ischemic encephalopathy that was designed to investigate whether outcomes would be improved by deeper (32 °C) or longer cooling (120 h), as compared with those achieved by standard whole body cooling (33.5 °C for 72 h). Using prior trial data, we developed neutral and enthusiastic prior probabilities for the effect on predischarge mortality, defined stopping guidelines for a clinically meaningful effect, and derived posterior probabilities for predischarge mortality.

**Results:**

Bayesian relative risk estimates for predischarge mortality were closer to 1.0 than were frequentist estimates. Posterior probabilities suggested increased predischarge mortality (relative risk > 1.0) for the three intervention groups; two crossed the Bayesian futility threshold.

**Conclusions:**

Bayesian analysis incorporating previous trial results and different pre-existing opinions can help interpret accruing data and facilitate informed stopping decisions that are likely to be meaningful and convincing to clinicians, meta-analysts, and guideline developers.

**Trial registration:**

ClinicalTrials.gov NCT01192776. Registered on 31 August 2010.

**Electronic supplementary material:**

The online version of this article (doi:10.1186/s13063-016-1480-4) contains supplementary material, which is available to authorized users.

## Background

Decisions to stop randomized trials are often based on traditional *P* values from sequential monitoring methods that diminish the possibility of making false positive claims of benefit or harm with repeated interim analyses [1, 2]. However, these decisions are often unconvincing to clinicians or guideline development panels [3]. The appropriate stopping guidelines are unclear [3–12], and the decisions are often difficult for data and safety monitoring committee (DSMC) members, who bear the responsibility for both avoiding recommendations to continue trials too long once harm or futility is suspected and for stopping trials too soon because of misleading interim findings. Safety concerns for patients enrolled in the current trial are self-evident in the former case. In the latter case, an erroneous conclusion that a truly beneficial therapy is ineffective or harmful can also be detrimental to a very large number of future patients, particularly if the therapy would in fact reduce rates of major adverse outcomes.

Bayesian methods for monitoring efficacy, safety, and futility have been proposed [13–24]. Bayesian approaches have potential advantages [25–27] that include incorporating the results from prior trials to better assess the likelihood of treatment benefit or harm and ensure that treatment recommendations are well justified, based on all relevant trials [16]. Another advantage of a Bayesian approach is that uncertainty from all parameter estimates is accounted for in reported summaries, which is particularly important with sparse data [28, 29]. For monitoring of trials, at a given interim analysis the posterior probability of the treatment effect is computed from the prior probability (referred to in this paper as “the prior”) and the interim data. Then DSMCs can weigh the current evidence for benefit, harm, or futility from the posterior probability and decide whether to continue recruitment, or to pause or terminate the trial.

A Bayesian approach can also incorporate a wide range of viewpoints and indicate the magnitude of the difference between treatment groups that would be needed at the end of the trial to convince those who are skeptical, as well as those who were enthusiastic about the value of the therapy prior to the trial [16, 30]. Decisions to stop a trial early based on the best available data from all relevant trials and on the identification of clinically meaningful differences are most likely to be convincing to meta-analysts, practice guideline developers, and clinicians.

Despite these advantages, only a small percentage of Phase III trials have adopted Bayesian monitoring methods, largely due to the seemingly daunting task of specifying priors [31], the perceived drawback of their subjective nature, computational burden [21], and lack of familiarity with implementation and interpretation of these methods [32, 33]. The objective of this report is to illustrate the use of a Bayesian approach and its advantages in trial monitoring with a concrete example of a major randomized trial of hypothermia in neonates with hypoxic-ischemic encephalopathy [34], a condition associated with a high risk of death or severe impairment. This trial used a frequentist monitoring plan and was stopped early for futility and safety concerns though stopping boundaries were not crossed. We present how Bayesian stopping guidelines can be specified using clinically meaningful treatment effects. We show how information from prior trials can be incorporated and utilized in addressing whether even an enthusiastic (about treatment benefit) clinician or investigator should be convinced by negative interim findings.

## Methods

### Optimizing Cooling Trial

Therapeutic hypothermia may be considered the most important advance in decades in the treatment of newborn infants with hypoxic-ischemic encephalopathy, a condition probably resulting from severe acute hypoxia-ischemia occurring within hours before birth [35, 36]. Whole body cooling to 33.5 °C for 72 h was shown to reduce the risk of death or neurodevelopmental impairment at 18–22 months of age by 28 % (relative risk, 0.72; 95 % confidence interval, 0.54–0.95) among term infants in a randomized trial conducted by the Eunice Kennedy Shriver National Institute of Child Health and Human Development (NICHD) Neonatal Research Network (NRN) [37]. However, even with cooling, only 56 % of the infants survived without severe or moderate impairment, and it was postulated that refined approaches to cooling would further improve the outcome. Based on evidence from studies on animals and neonates [38–42], the NRN launched a trial (Optimizing Cooling Trial) in 18 centers to assess whether the use of deeper cooling (to 32 °C), longer cooling (for 120 h), or both would further increase survival without impairment over that achieved with standard cooling [34–36].

#### Study design

The Optimizing Cooling Trial utilized a 2 × 2 factorial design to test depth and duration of cooling. Infants of 36 weeks gestational age or older with severe acidosis or need for resuscitation at birth with moderate or severe hypoxic-ischemic encephalopathy were randomized to four hypothermia groups: 33.5 °C for 72 h, 32.0 °C for 72 h, 33.5 °C for 120 h, or 32.0 °C for 120 h. Randomization was stratified by center and severity of hypoxic-ischemic encephalopathy (moderate or severe) with a dichotomous composite primary outcome of death or moderate or severe disability at 18–22 months of age. The sample size calculation was based on frequentist marginal analyses of the two cooling factors (comparing 33.5 °C with 32 °C and 72 h with 120 h) and assumed that there was no large interaction between duration and depth of cooling. The trial planned to enroll a total of 726 infants to detect the hypothesized relative risk (RR) of 0.73 in either factor with expected primary outcome rates of 37.5 % and 27.5 % for the two marginal groups with 80 % power and a two-sided *α* of 0.05 (Table 1). A completed CONSORT 2010 checklist is provided in Additional file 1.

**Table 1:** Hypothesized rates of primary outcome of death or moderate or severe impairment at 18–22 months: these rates were used for sample size calculation

	Depth of cooling		
	33.5 °C	32.0 °C	Margin
Duration of cooling			
72 h	45 %	30 %	37.5 %
120 h	30 %	25 %	27.5 %
Margin	37.5 %	27.5 %	

#### Ethical considerations

The research study was approved by the local institutional review board of the Women and Infants Hospital of Rhode Island, Case Western Reserve University, the Children’s Mercy Hospital, the University of Cincinnati Medical Center, the Cincinnati Children’s Hospital Medical Center, the Good Samaritan Hospital, the Duke University School of Medicine, the University of North Carolina at Chapel Hill, Emory University, Grady Memorial Hospital, Indiana University, the Nationwide Children’s Hospital, RTI International, Stanford University, the University of Alabama at Birmingham Health System, the University of California—Los Angeles, the University of Iowa, Mercy Medical Center, the University of New Mexico Health Sciences Center, the University of Pennsylvania, the University of Texas Southwestern Medical Center at Dallas, the University of Texas Health Science Center at Houston, Wayne State University, the University of Michigan Medical Center, the University of Rochester Medical Center, and the University of Buffalo Women’s and Children’s Hospital of Buffalo. Written informed consent was obtained from a parent or guardian for each enrolled infant.

#### Trial monitoring

The trial was monitored for safety outcomes of cardiac arrhythmia, persistent acidosis, major vessel thrombosis, alteration of skin integrity, major bleeding, and death using Pocock boundaries constructed to maintain an overall *α* of 0.05 for each outcome. Prespecified safety interim analyses were planned after the first 50 infants were enrolled and then for every 25 infants thereafter. The results of each interim analysis were reviewed by the independent DSMC. Though not specified in the study protocol, futility analyses of predischarge mortality were also performed at the request of the DSMC during the final interim check. These marginal analyses (comparing two cooling factors) were performed by calculating conditional power using the hypothesized treatment effect for the primary outcome as the alternative hypothesis.

#### Interim analysis using frequentist approaches

Enrollment started in October 2010. Following the recommendation of the DSMC, the NICHD Director stopped the trial for concerns about safety and futility on 27 November 2013, after the eighth DSMC review. A total of 364 infants had been enrolled (Additional file 2). The observed rates of predischarge mortality are shown in Table 2. The RR (95 % confidence interval) for predischarge mortality, adjusted for level of hypoxic-ischemic encephalopathy and center, was 1.37 (0.92–2.04) for the duration of cooling comparison, and 1.24 (0.69–2.25) for the depth of cooling comparison. Although the data did not cross the stopping boundaries for safety, the conditional power of 2 % for both marginal comparisons indicated a low probability of finding a statistically significant reduction in predischarge deaths were the study to continue to completion.

**Table 2:** Observed rates of predischarge mortality for the Optimizing Cooling Trial

	Depth of cooling		
	33.5 °C	32.0 °C	Margin
Duration of cooling			
72 h	7 % (7/95)	14 % (13/90)	11 %
120 h	16 % (15/96)	17 % (14/83)	16 %
Margin	12 %	16 %	

Data on the primary outcome of death or moderate or severe disability at 18 to 22 months were available for only a few infants and hence did not play a role in the decision to stop the trial, a reason that some observers might question this decision. Moreover, the predischarge mortality with standard cooling (7 %) in the Optimizing Cooling Trial was less than half that in a prior NRN trial (19 %) using the same eligibility criteria [37]—and the mortality rates for all three experimental groups (longer cooling, deeper cooling, both) were less than 19 %. Thus, the very low mortality in the standard group might be a “random low” as in other trials when the interim findings after an equal or larger number of patients proved to be quite misleading [3, 4, 43, 44]. However, this low mortality might be partly or fully due to improvements in care or changes in the patient population between the two trials, especially considering the 7 year gap between them. Compared with cooled infants in the prior NRN trial, infants in the Optimizing Cooling Trial were less likely to have severe hypoxic-ischemic encephalopathy (23 % versus 32 %), to be intubated at birth (79 % versus 95 %), or to have seizures (29 % versus 43 %).

#### What could a Bayesian monitoring approach add to an interim analysis?

In developing stopping guidelines, data from the prior NRN trial could be used in identifying what negative interim findings for predischarge mortality would be convincing to enthusiasts as well as skeptics or neutral clinicians. While no prior data exist for longer or deeper cooling, the observed proportion of predischarge mortality for the cooled group in the prior trial can inform the expected rate for the standard group in the Optimizing Cooling Trial, as well as realistic treatment effects for this outcome.

Another advantage of a Bayesian approach is that it forces investigators to consider carefully what posterior probability of benefit or harm would justify stopping the trial—an exercise that requires close collaboration between clinical investigators and biostatisticians. The appropriate stopping probability threshold should arguably be lower for treatment harms than benefits, meaning that less evidence might be required for presence of harm than absence of benefit to stop a trial. Moreover, a particularly high probability of benefit might be required for therapies that are invasive, hazardous, or extremely expensive. Once the threshold posterior probabilities have been selected, simulations can be performed if necessary to satisfy regulatory agencies or verify acceptable frequentist characteristics (type I error, power) [21, 26, 45].

While the *P* values required by frequentist stopping guidelines can be modified based on these considerations, this is rarely done in practice. Bayesian stopping guidelines based on these considerations may thus be more flexible as well as more easily explained and more meaningful to clinicians and developers of practice guidelines. They also have the added benefit of forcing difficult but productive upfront discussions among clinical investigators and biostatisticians that can enrich all aspects of trial design.

### Bayesian monitoring of the Optimizing Cooling Trial

In addition to the necessary elements of an acceptable data monitoring plan [46], three main components need to be specified for a Bayesian monitoring plan: (1) prior evidence (in some circumstances, expert opinion might also be considered) of treatment effect; (2) clinically important treatment effect(s) for the primary outcome and any important outcomes, including death, to be monitored; and (3) probability thresholds for stopping a trial (Table 3). We illustrate how these three components can be specified in practice using the Optimizing Cooling Trial as an example.

**Table 3:** Summary of key components of a Bayesian monitoring plan

Component	Specification	Example
Prior distributions	• Previous studies on the control rate or treatment effect can be used as prior information • Prior beliefs about the treatment effect should be elicited from experts to inform the strength of the evidence needed to convince them	Two-arm trial of Treatment A versus B (control): • Evidence from three previous trials on rate of outcome under treatment B: 17 %, 25 %, 30 % • Evidence from two studies on treatment effect for different population: RR 0.98 (95 % CI: 0.73–1.3); RR 0.75 (95 % CI: 0.56–1.0) Prior distributions, center (95 % CrI): • Control rate: 25 % (5–55 %) • Skeptical prior for treatment effect: RR 1.10 (0.7–2.0) • Enthusiastic prior for treatment effect: RR 0.85 (0.5–1.0)
Clinically important treatment effect	• Investigators should specify how big a treatment effect needs to be in order to stop a trial and recommend its use or advise against it	• A relative risk reduction of 15 % or more is needed to recommend treatment A, RR < 0.85 • An absolute increase of 2 % in safety outcome would be unacceptable, RD > 0.02
Stopping thresholds	• For each type of monitoring, i.e., safety, efficacy, or futility, the level of confidence to stop the trial early needs to be specified • For most cases, it should be based on a clinically important effect • Efficacy: the trial will stop early if the likelihood of seeing a clinically important effect is very large, even under a skeptical prior • Futility: if the likelihood of a clinically important effect is small even under an enthusiastic prior, the trial would stop early • Safety: the trial would stop if the probability of increasing harm is large enough under an enthusiastic prior	At any preplanned interim analysis, any of these occurrences would make the DSMC consider stopping the trial: • Efficacy under skeptical prior: Pr(RR < 0.85) > 0.99 • Futility under enthusiastic prior: Pr(RR < 0.85) < 0.10 • Safety under enthusiastic prior: Pr(RD > 0.02) > 0.70

*DSMC*, data and safety monitoring committee, *CI*, confidence interval, *CrI* credible interval, *Pr*, probability, *RD* risk difference, *RR*, relative risk

We performed a Bayesian reanalysis of predischarge mortality data from the 364 infants enrolled in the Optimizing Cooling Trial. We present posterior probabilities for the comparisons of the three hypothermia groups with standard cooling partly because the study protocol stated the possibility of terminating one or more groups for safety or futility and continuing the trial with the remaining groups. This approach of simultaneously monitoring all groups for futility was found to be superior to or as good as an approach that first assesses an interaction term between the interventions and then examining the main effects if no significant interaction is found [47]. To compare with the frequentist results, we also provide posterior probabilities for the marginal comparisons of longer cooling or deeper cooling.

#### Bayesian model

We used a binomial model with a log link to estimate the RR of predischarge mortality with longer cooling, deeper cooling, or both, compared with standard cooling. We included the main effects of duration and depth of cooling and the interaction term to assess its magnitude. Letting *y*_*i*_ be the outcome of predischarge death (1 = yes, 0 = no), the model is expressed as:

$$ {y}_i \sim \mathrm{Bernoulli}\left({p}_i\right), $$

$$ \log \left({p}_i\right)={\beta}_0+{\beta}_1\ {\mathrm{depth}}_i+{\beta}_2\ {\mathrm{duration}}_i+{\beta}_3\ {\mathrm{depth}}_i\times {\mathrm{duration}}_i, $$

where *p*_*i*_ is the probability of predischarge death for infant *i*, depth and duration are coded as 1 for 32.0 °C and 120 h (experimental interventions) and 0 otherwise, and *β* are regression coefficients. In the log RR scale, the marginal effects of depth and duration are given by *β*_1_ + *β*_3_/2 and *β*_2_ + *β*_3_/2, respectively, with negative values of *β* indicating decreased mortality. *β*_0_ is the log probability of predischarge death for the standard cooling group. The effects of each of the three intervention groups compared with standard cooling are *β*_1_ for deeper cooling given alone; *β*_2_ for longer cooling given alone; and *β*_1_ + *β*_2_ + *β*_3_ when both therapies are given. Thus, this model easily allows for the estimation of the marginal treatment effect of the two factors as well as individual treatment group comparisons [48]. We did not include center or level of hypoxic-ischemic encephalopathy variables in our analysis.

While this model gives direct estimates of RRs, it is straightforward to derive estimates for other risk measures, such as absolute risk difference (RD) or its reciprocal, the number needed to treat (see Additional file 3).

#### Prior distributions

We assumed independent normal prior distributions for the *β* regression coefficients: *β*_0_ ~ normal(*μ*_0_, *τ*_0_^2^), *β*_1_ ~ normal(*μ*_1_, *τ*_1_^2^), *β*_2_ ~ normal(*μ*_2_, *τ*_2_^2^), and *β*_3_ ~ normal(*μ*_3_, *τ*_3_^2^), where the *μ* and *τ* are, respectively, the means and standard deviations of the distributions. We specified a set of neutral prior distributions and a set of enthusiastic priors for the two marginal interventions. With both sets of priors, we assumed an expected predischarge mortality rate of 19 % for the standard cooling group, the observed rate in the previous NRN trial. The prior for *β*_0_ has a mean of log(0.19) = −1.66 and a standard deviation of 0.565, which increases the uncertainty observed in the previous NRN trial (0.19 in log scale) by a factor of three (to account for population differences between the two cooling trials).

#### Neutral priors

We centered the RR at 1.0 (mean of 0 in the log RR scale), indicating no *a-priori* difference between the treatments being compared, and used a 95 % credible interval (CrI; this interval is interpreted as having a 95 % probability of containing the true RR) of 0.33–3.0. While empirical evidence from Cochrane systematic reviews of neonatal studies [49] indicates that the great majority of observed treatment effects on mortality are in the range of 0.5–2.0, there was very little previous information on the two interventions being tested, and we broadened this interval to allow for the possibility of greater harm or benefit. The implied neutral priors for the *β* coefficients have means of *μ*_0_ = −1.66 and *μ*_1_ = *μ*_2_ = *μ*_3_ = 0 and standard deviations of *τ*_0_ = *τ*_1_ = *τ*_2_ = 0.565 and *τ*_3_ = 0.14. The prior standard deviation for *β*_3_ indicates an *a-priori* probability of 0.025 of a qualitative interaction between longer and deeper cooling (meaning that the effect of longer cooling on the outcome changes direction in the presence or absence of deeper cooling). This corresponds to specifying that the likelihood of reducing the relative risk by 24 % with longer and deeper cooling (RR = 0.76) is only 0.025 when the treatment effect is zero (RR = 1) in the presence of only one of the interventions (i.e., Pr[*β*_3_ < log(0.76)|*β*_1_, *β*_2_ = 0] = 0.025) [50]. For sensitivity analysis, we set *τ*_3_ = 0.408, which corresponds to a 0.25 *a-priori* probability of a qualitative interaction [48].

#### Enthusiastic priors

Predischarge death rates with cooling were available only from the prior multicenter NRN trial. Based largely on this trial, we chose to center the enthusiastic priors at a treatment effect half the size of the hypothesized reductions for the primary outcome (death or impairment at 18–22 months). We used the same standard deviations as for the neutral prior. For the two marginal effects, we centered the prior at a RR of 0.85 (assumed rates of 16 % for longer cooling or deeper cooling and 19 % for standard cooling). The implied priors for the *β* coefficients have means *μ*_0_ = −1.66, *μ*_1_ = *μ*_2_ = −0.1625, and *μ*_3_ = 0. The prior for *β*_3_ is again centered at 0, since there is no prior expectation of an interaction, with a small *a-priori* probability (0.025) of a qualitative interaction between longer and deeper cooling.

**Fig. 1:**
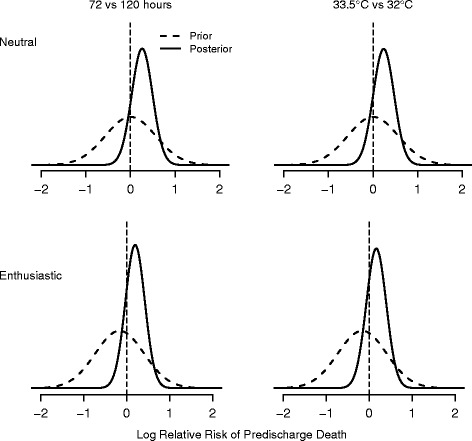
Probabilities of treatment benefit (log RR) for marginal comparisons of cooling on predischarge mortality. Negative values favor the experimental group. *Left panel* shows the marginal duration comparison (*β*_2_ + *β*_3_/2) and the *right panel* the marginal depth comparison (*β*_1_ + *β*_3_/2). Top (*bottom*) panel shows the neutral (enthusiastic) prior and corresponding posterior for the two-factor marginal comparisons

**Fig. 2:**
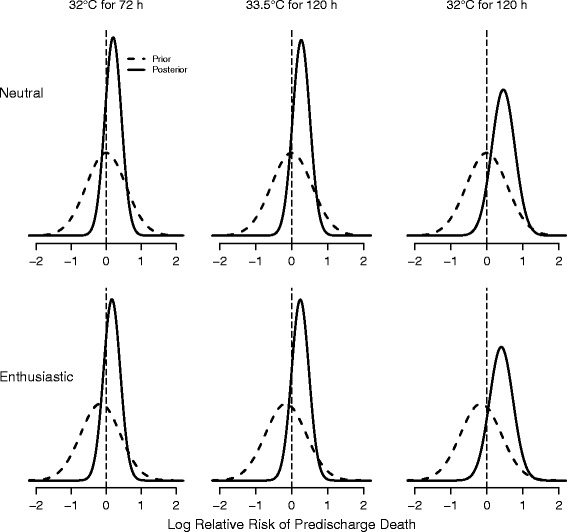
Probabilities of treatment benefit (log RR) on predischarge mortality for three experimental cooling groups. Negative values favor the experimental group. Deeper cooling (*β*_1_; *left panel*), longer cooling (*β*_2_; *middle panel*), and both (*β*_1_ + *β*_2_ + *β*_3_; *right panel*) are compared with standard cooling (33.5 °C for 72 h). Top (*bottom*) panel shows the neutral (enthusiastic) prior and corresponding posterior probabilities

Implied priors for the marginal comparisons and for the three cooling groups compared with standard cooling are shown in Figs. 1 and 2, respectively.

#### Interim monitoring for futility

We defined a futility stopping guideline for predischarge mortality based on a clinically meaningful treatment effect size [22, 24]. If the posterior probability of this clinically meaningful mortality reduction dropped below a prespecified threshold, the DSMC would consider terminating the trial. Suppose the investigators had decided when designing the trial that the interventions had to reduce predischarge mortality by 10 % or more, meaning a RR < 0.90 (or an absolute RD of 2 %). If the probability of one of the cooling groups reducing predischarge mortality by at least 10 % fell below 0.10,

$$ \Pr \left(\mathrm{R}\mathrm{R} < 0.90\Big|\mathrm{Interim}\ \mathrm{data}\right) < 0.10, $$

the cooling group would be stopped for futility. If all groups met this threshold, the trial would be stopped.

Suppose that instead of a reduction in RR, the trial investigators were interested in an absolute RD, defined as *p*_control_ − *p*_treatment_, of at least 1 %. While a 1 % absolute difference in mortality has been considered a small effect in neonatal trials, differences of this magnitude for death or for composite outcomes of death or major cardiovascular events have been considered sufficiently important to justify treatment recommendations from adult trials [51–55]. With the number of disability free life years at stake in a neonatal trial, a 1 % difference in mortality or death or disability would likewise be important. The stopping guideline was that a hypothermia group would be stopped for futility if the probability of a RD of 1 % or more fell below 0.10,

$$ \Pr \left(\mathrm{R}\mathrm{D} > 0.01\Big|\mathrm{Interim}\ \mathrm{data}\right) < 0.10. $$

#### Interim monitoring for safety

As another example of what the investigators might have selected as a stopping guideline in designing the trial, enrollment in any intervention group could be stopped if the probability of a 5 % absolute increase in mortality (the RD being less than negative 5 %) were 50 % or greater,

$$ \Pr \left(\mathrm{R}\mathrm{D} < -0.05\Big|\mathrm{Interim}\ \mathrm{data}\right) > 0.50. $$

Acceptable operating characteristics would need to be verified before using these thresholds [26].

#### Implementation details

All models were fitted via Markov chain Monte Carlo (MCMC) methods, since the posterior distribution of the parameters is not in closed form [56]. We constrained all *p*_*i*_ < 1 in the models. All analyses were conducted using WinBUGS 1.4.3 [57]. For each model, we used three MCMC chains, each with 40,000 iterations, in addition to an initial burn-in of 4,000 chains. We examined trace plots of all the parameters to monitor convergence and calculated the Gelman–Rubin diagnostic $\widehat{R}$. Additional file 3 gives the sample code for fitting this log binomial model in WinBUGS, and shows how to calculate RRs and RDs from the model parameters for both the two-factor marginal comparisons and the three intervention groups.

## Results

For all models, trace plots showed good mixing of the 3 MCMC chains and the $\widehat{R}$ < 1.01 for all parameters, indicating convergence.

### Marginal comparisons of longer cooling and deeper cooling

Under neutral priors, the posterior distribution of the RR for longer cooling has a median of 1.30, (95 % CrI, 0.82–2.04) and an 87 % probability of increased predischarge mortality with longer duration (Fig. 1, top left panel). Deeper cooling has an 81 % posterior probability of increased predischarge death compared with standard cooling (RR: posterior median, 1.22; 95 % CrI, 0.77–1.87; Fig. 1). The likelihood of the RR < 0.90 given the interim trial data is 6 % and 9 % for longer cooling and deeper cooling, respectively, which are below the 10 % futility stopping threshold. Using the risk difference for monitoring, the probability of a RD > 1 % (indicating reduced mortality of 1 % or more with intervention) is 12 % for deeper cooling and 8 % for longer cooling, and longer cooling would be stopped.

Figure 1 (bottom panels) shows the posterior distributions calculated from the enthusiastic priors. The posterior median for the RR for duration of cooling is 1.27 (95 % CrI, 0.81–2.01) with 84 % posterior probability of increased predischarge death with longer cooling. Compared with standard cooling, deeper cooling has 76 % probability of increased predischarge mortality (RR: posterior median, 1.18; 95 % CrI, 0.74–1.85). The probability of a 10 % or greater reduction in predischarge mortality (RR < 0.90) is 7 % (9 % for RD >1 %) and 12 % (15 % for RD >1 %) for the duration and depth interventions. Longer cooling again crosses the futility stopping threshold.

### Posterior distributions for three intervention group comparisons

Table 4 and Fig. 2 give posterior summaries for the RRs of the three cooling groups (longer cooling, deeper cooling, both) compared with standard cooling. The posterior medians for all three groups are above 1.0, indicating increased mortality compared with standard cooling under both neutral and enthusiastic priors. Under the neutral prior, the probability of any reduced mortality is small for the 32 °C for the 120 h group. However, an enthusiastic prior gives a 25 % probability of reduced mortality for the 32 °C for 72 h group. The probabilities of treatment benefit of the three experimental groups achieving a clinically meaningful value of RR < 0.90 are 13 %, 8 %, and 6 % under an enthusiastic prior and somewhat smaller under the neutral prior. Applying the same futility criterion as for the marginal interventions, only the 32 °C for 72 h group would not meet the stopping threshold under the enthusiastic prior. Similarly, using the futility stopping guideline based on a minimum RD of 1 %, the 33.5 °C for 120 h and 32 °C for 120 h groups would be stopped with the interim data (Table 5). Finally, only the 32 °C for 120 h group crosses the safety stopping threshold for predischarge death.

**Table 4:** Summaries of posterior probabilities of relative risk of predischarge mortality

	RR posterior median (95 % credible interval)		Evidence of any benefit Pr(RR < 1.0)		Futility monitoring Pr(RR < 0.90)	
	Neutral	Enthusiastic	Neutral	Enthusiastic	Neutral	Enthusiastic
32.0 °C for 72 h	1.23 (0.76–1.92)	1.19 (0.74–1.87)	20 %	25 %	10 %	13 %
33.5 °C for 120 h	1.31 (0.82–2.09)	1.27 (0.80–2.03)	13 %	16 %	6 %	8 %
32.0 °C for 120 h	1.60 (0.82–2.97)	1.50 (0.79–2.83)	8 %	11 %	4 %	6 %

The three experimental hypothermia groups are compared with standard cooling (33.5 °C for 72 h) under a neutral and enthusiastic prior. RR values less than 1.0 favor experimental groups

*PR* probability, *RR* relative risk

**Table 5:** Summaries of posterior probabilities of the absolute risk difference of predischarge mortality

	RD posterior mean (95 % credible interval)		Futility monitoring Pr(RD > 0.01)^a^		Safety monitoring Pr(RD < −0.05)^b^	
	Neutral	Enthusiastic	Neutral	Enthusiastic	Neutral	Enthusiastic
32.0 °C for 72 h	−0.02 (−0.08, 0.03)	−0.02 (−0.08, 0.04)	11 %	15 %	19 %	16 %
33.5 °C for 120 h	−0.03 (−0.09, 0.02)	−0.03 (−0.09, 0.03)	8 %	9 %	28 %	25 %
32.0 °C for 120 h	−0.06 (−0.15, 0.03)	−0.06 (−0.15, 0.03)	5 %	7 %	61 %	54 %

^a^RD > 0.01 indicates 1 % or more reduced mortality

^b^RD < −0.05 indicates a 5 % or more absolute increase in mortality

The three experimental groups are compared standard cooling (33.5 °C for 72 h) under a neutral and enthusiastic prior. Positive values of RD favor the experimental groups

*Pr* probability, *RD* risk difference

Sensitivity analyses using a larger standard deviation for the prior distribution of the interaction term resulted in similar posterior probabilities (not shown) with no differences in the crossing of stopping thresholds.

## Discussion

The use of the best available prior information is one of the main advantages of the Bayesian approach, as it allows for formal evaluation of all available evidence. While no prior data exist for longer or deeper cooling, we explain how the data from a prior NRN trial of standard cooling [37] could be used to identify what negative interim findings for predischarge mortality should be convincing to enthusiasts as well as skeptics or neutral clinicians. The Bayesian analyses presented here incorporated this information into the prior probabilities while excluding large treatment effects, which are almost never observed with clinical interventions [58, 59]. The resulting posterior estimates of the RR being closer to 1.0 than the unadjusted frequentist estimates (e.g., 1.30 versus 1.50 for duration of cooling) even under a neutral prior.

These Bayesian interim analyses illustrate how the Bayesian approach can be used by DSMCs to evaluate the “totality of available evidence” [60] when deciding whether to stop a trial early. By using enthusiastic priors, a DSMC can judge whether the current evidence should be sufficient to convince an investigator with a strong prior belief in treatment benefit that there is little chance of benefit from the intervention. Under our proposed priors and stopping guidelines based on RRs and focusing on marginal comparisons, the trial data would not support the existence of treatment benefit for longer cooling but would not completely rule out the existence of a clinically important benefit (12 % probability) for deeper cooling.

We conducted a supplementary analysis using a neutral prior (centered at 0) for the intercept (and the same neutral priors for all other parameters) that essentially ignored the evidence on the rate of predischarge mortality from the previous NRN trial. The results are given in Additional file 3 and show RR estimates that are further away from 1.0 (indicating less shrinking towards a null effect). Under this prior, all three experimental groups cross the futility stopping threshold. Other priors not presented here, such as a skeptical prior (centered at RR > 1) or robust priors (e.g., Student’s *t* distributions) for sensitivity analyses, could also be presented to give DSMC members and investigators a complete picture of pre-existing expert views of a therapy’s benefits and harms. While the subjectivity of the prior is usually seen as the biggest drawback of a Bayesian approach, we see it as an advantage, since it formalizes how experts with differing pre-existing opinions will view the results. A DSMC can then consider whether the results of a trial would be convincing to the whole community.

When factorial designs are used, the possibility of dropping one or two treatment groups and continuing the trial with the remaining groups should be discussed before starting the trial. Here we illustrated Bayesian monitoring for the three experimental cooling groups. The results show that the futility stopping threshold based on RRs and RDs would have been crossed for the 32.0 °C for 120 h and 33.5 °C for 120 h groups under both priors. However, the 32.0 °C for 72 h cooling group would not have crossed the threshold under either prior. Of course, different stopping thresholds would lead to different decisions. For example, if we instead used a RD of ≥5 % to monitor futility, the posterior probabilities of this treatment effect, Pr(RD > 0.05), would be less than 2 % for all three groups, even with the enthusiastic prior. These results illustrate the need to fully explore at the planning stage the choice of priors and relevant quantities to monitor for efficacy, safety, and futility, as well as the probability thresholds for stopping the trial. All aspects of the trial design and monitoring plan can be evaluated with simulation studies (using different scenarios to represent a range of potential treatment effects) to ensure adequate type I and II errors [26, 45]. As with frequentist monitoring rules, planned Bayesian interim analyses and stopping guidelines should be prespecified in the protocol [61].

A frequentist interim futility analysis using conditional power (the probability of obtaining a statistically significant benefit given current interim data) calculations led to the recommendation to stop this trial. A disadvantage of this approach is that it might put too much focus on an arbitrary level of significance [7] or a secondary outcome. A high probability of not showing a statistically significant benefit is not necessarily sufficient reason to stop a trial, since significance may not be needed to justify recommendation of a therapy with at least equivalent benefit that is less invasive, hazardous, more convenient, or less expensive than the comparison therapy. With the overriding importance of death and of death or disability, clinicians and their patients and families might consider a lower rate of death or of death or disability for the interventions assessed in the Optimizing Cooling Trial to justify their use, even if the differences did not reach statistical significance.

A Bayesian futility monitoring plan could alternatively use the predictive probability of a successful trial at the end of planned enrollment. This predictive probability is analogous to the frequentist conditional power but accounts for the uncertainty of the current parameter values rather than assuming fixed values. However, we would argue that it suffers from the same limitations as conditional power. Although as Saville et al. [21] and Emerson et al. [62] point out, any stopping rule based on the posterior distribution can be converted into a stopping rule based on the predictive probability. For the Optimizing Cooling Trial, lack of primary outcome data also precluded us from adopting a predictive probability approach.

Challenges to the wider use of Bayesian monitoring methods include the perceived subjectivity of this approach, the difficulty of eliciting priors from investigators and previous studies, computational complexities, and reluctance from funding agencies and journals to embrace Bayesian methods in clinical research. Statisticians and clinical investigators must collaborate to find ways of overcoming these barriers to best inform decision makers. For the Optimizing Cooling Trial, we prespecified and planned to conduct a Bayesian final analysis for the primary outcome of death or disability.

## Conclusions

To help familiarize clinical investigators with Bayesian monitoring methods, we reanalyzed the Optimizing Cooling Trial, a neonatal trial that was stopped early for safety and futility. We incorporated information on predischarge mortality rate from a previous multicenter neonatal cooling trial into the prior distribution. When we incorporate external data and take the view of an enthusiast, two of the three intervention groups would be stopped for futility.

Bayesian analyses incorporating previous trial results and different pre-existing opinions can help interpret accruing data and facilitate informed stopping decisions likely to be meaningful and convincing to clinicians, meta-analysts, and guideline developers. Given the advantages of Bayesian trial monitoring, investigators should consider the use of Bayesian methods in Phase III clinical trials.

## Abbreviations

CrI, credible interval; DSMC, data and safety monitoring committee; MCMC, Markov chain Monte Carlo; NICHD, Eunice Kennedy Shriver National Institute of Child Health and Human Development; NRN, Neonatal Research Network; RD, absolute risk difference; RR, relative risk

## Additional files

Additional file 1: CONSORT 2010 checklist. (PDF 677 kb) Additional file 2: CONSORT flow diagram. (PDF 37 kb) Additional file 3: Additional analysis results and sample WinBUGS code. (PDF 132 kb)
