# Supplementary figures and images for: Advantages of Bayesian monitoring methods in deciding whether and when to stop a clinical trial: an example of a neonatal cooling trial
Source: Trials. 2016 Jul 22;17:335. doi: 10.1186/s13063-016-1480-4 (PMC4957277; doi:10.1186/s13063-016-1480-4)

Figure A1: Enrollment of neonates in the Optimizing Cooling Trial

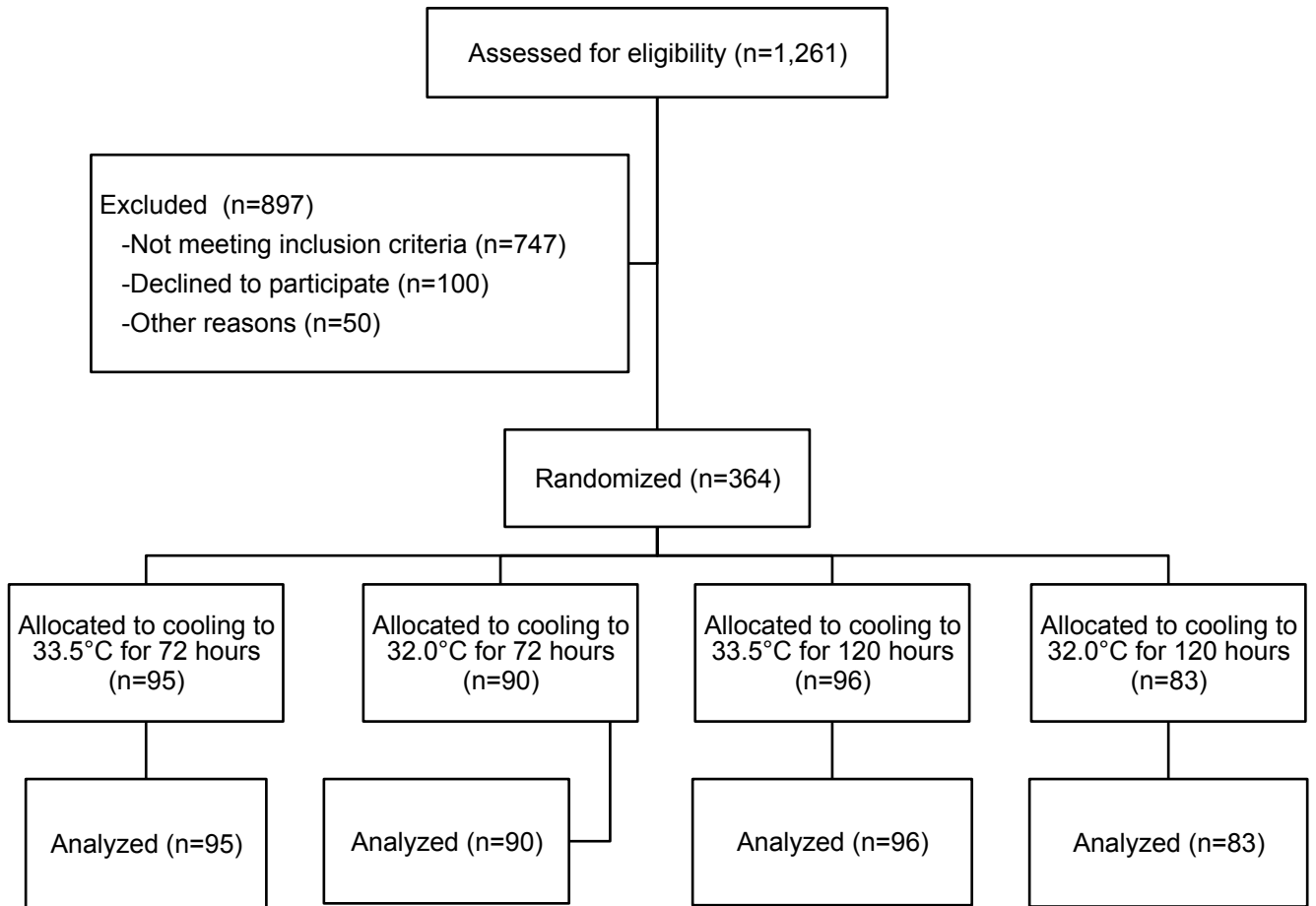

Supplement: Additional file 2: — CONSORT flow diagram. (PDF 37 kb) [file 13063_2016_1480_MOESM2_ESM.pdf]
