# Supplementary material for: Advantages of Bayesian monitoring methods in deciding whether and when to stop a clinical trial: an example of a neonatal cooling trial
Source: Trials. 2016 Jul 22;17:335. doi: 10.1186/s13063-016-1480-4 (PMC4957277; doi:10.1186/s13063-016-1480-4)
Supplement: Additional file 3: — Additional analysis results and sample WinBUGS code. (PDF 132 kb) [file 13063_2016_1480_MOESM3_ESM.pdf]

Additional File for 'Advantages of Bayesian monitoring methods in deciding whether and when to stop a clinical trial: an example of a neonatal cooling trial'

Results under neutral, mildly informative priors for all parameters.

We conducted a supplementary analysis using priors centered at 0 for all parameters. Specifically, we used a Normal(0, 10^2) for the intercept β_0 and the same Normal(0, 0.565^2) for β_1, β_2, and β_3 that imply a 95% prior interval of 0.33-3.0 in the RR scale. Summaries of the posterior distributions for the 3 experimental groups compared to standard cooling are shown in Table A1.

Table A1. Summaries of posterior probabilities of relative risk of predischarge mortality. The 3 experimental hypothermia groups are compared to standard cooling (33.5°C for 72 hours) under a neutral prior. RR values less than 1.0 favor experimental groups.

	RR Posterior Median (95% CrI)	Evidence of Any Benefit Pr(RR < 1.0)	Futility Monitoring Pr(RR < 0.90)
32.0°C for 72 hours	1.35 (0.76-2.4)	15%	8%
33.5°C for 120 hours	1.45 (0.82-2.6)	10%	5%
32.0°C for 120 hours	1.76 (0.88-3.5)	6%	3%

Sample code

Sample WinBUGS code to fit a log Binomial Bayesian model for a 2 X 2 factorial trial incorporating informative prior information for the intercept. Model 1 uses neutral priors for the intervention main effects denoted by `b1` and `b2`. Model 2 uses enthusiastic priors for `b1` and `b2`.

A1. Model 1

```
model
{
      for(i in 1:N){
      y[i]~dbin(pi[i],1)
      log(pi[i])<-b0+b1*depth[i]+b2*duration[i]+b3*depth[i]*duration[i]
      }

      #Constraints all probabilities pi[] to be less than 1.
      for (i in 1:364){
      ones[i] <- 1
      ones[i] ~ dbern(C1[i])
      C1[i] <- step(1-pi[i])
      }
```

```
#Priors
#Informative prior for intercept
b0~dnorm(-1.66, 3.13)

#Assumes neutral prior with credible intervals of 0.33-3.0 for RR
b1~dnorm(0, 3.13)
b2~dnorm(0, 3.13)

#Prior for interaction term assuming a very small probability
#(0.025) of a qualitative interaction
b3~dnorm(0,51)  ######## SD=0.14

#Prior below was used for sensitivity analyses on the interaction
#term, assumes probability of 0.25 of a qualitative interaction
#b3~dnorm(0,6)  ######## SD=0.408

##Probabilities of death in each group
p1<- exp(b0)
p2<- exp(b0+b1)
p3<- exp(b0+b2)
p4<- exp(b0+b1+b2+b3)

##Marginal probabilities of death for duration factor
pdur.72<-exp(b0+b1*mean(depth[]))
pdur.120<-exp(b0+b1*mean(depth[])+b2+b3*mean(depth[]))

##Marginal probabilities of death for depth factor
pdep.33<-exp(b0+b2*mean(duration[]))
pdep.32<-exp(b0+b1+b2*mean(duration[])+b3*mean(duration[]))


##Relative risks for marginal comparisons of duration and depth
RR.dur<-exp(b2+b3/2)
RR.dep<-exp(b1+b3/2)

##Relative risks for 3 hypothermia groups compared to standard
#cooling
RR1<-exp(b1)
RR2<-exp(b2)
RR3<-exp(b1+b2+b3)

##Risk differences for 3 hypothermia groups
RD1<-p1-p2
RD2<-p1-p3
```

```
    RD3<-p1-p4

    ## Probability of any benefit for 3 hypothermia groups compared
    #to standard
    pRR1.1<-1-step(b1)
    pRR2.1<-1-step(b2)
    pRR3.1<-1-step(b1+b2+b3)

    ## Probability of RR < 0.9 for 3 hypothermia groups compared to
    #standard
    pRR1.90<-step(-0.1053605-b1)
    pRR2.90<-step(-0.1053605-b2)
    pRR3.90<-step(-0.1053605-(b1+b2+b3))

    ###Probability of increased mortality by 5% or more for 3
    #hypothermia groups compared to standard

    pRD1.INC05<-step(p2-p1-.05)
    pRD2.INC05<-step(p3-p1-.05)
    pRD3.INC05<-step(p4-p1-.05)

    ###Probability of decreased mortality by 1% or more for 3
    #hypothermia groups compared to standard
    pRD1.DEC01<-step(p1-p2-.01)
    pRD2.DEC01<-step(p1-p3-.01)
    pRD3.DEC01<-step(p1-p4-.01)

    ##Probabilities of any benefit and RR < 0.9 for marginal
    #comparisons
    pRR.dur.1<-1-step(b2+b3/2)
    pRR.dur.90<-step(-0.1053605-(b2+b3/2))

    pRR.dep.1<-1-step(b1+b3/2)
    pRR.dep.90<-step(-0.1053605-(b1+b3/2))

    ##Probabilities of RD>0.01 and RD <(-0.05) for marginal
    #comparisons

    pRD.dur.INC05<-step(pdur.120-pdur.72-.05)
    pRD.dep.INC05<-step(pdep.32-pdep.33-.05)

    pRD.dur.DEC01<-step(pdur.72-pdur.120-.01)
    pRD.dep.DEC01<-step(pdep.33-pdep.32-.01)

}
```

A2. Model 2

```
model
{
	for(i in 1:N){
	y[i]~dbin(pi[i],1)
	log(pi[i])<-b0+b1*depth[i]+b2*duration[i]+b3*depth[i]*duration[i]
	}

	#Constraints all probabilities pi[] to be less than 1.
	for (i in 1:364){
	ones[i] <- 1
	ones[i] ~ dbern(C1[i])
	C1[i] <- step(1-pi[i])
	}

	#Priors
	#Informative prior for intercept
	b0~dnorm(-1.66, 3.13)

	#Enthusiastic priors with same SD as neutral priors
	b1~dnorm(-0.1625189, 3.13)
	b2~dnorm(-0.1625189, 3.13)

	b3~dnorm(0,51)  ######## SD=0.14

	#Prior below was used for sensitivity analyses on the interaction
	#term
	#b3~dnorm(0,6)  ######## SD=0.408

	##Probabilities of death in each group
	p1<- exp(b0)
	p2<- exp(b0+b1)
	p3<- exp(b0+b2)
	p4<- exp(b0+b1+b2+b3)

	##Marginal probabilities of death for duration factor
	pdur.72<-exp(b0+b1*mean(depth[]))
	pdur.120<-exp(b0+b1*mean(depth[])+b2+b3*mean(depth[]))

	##Marginal probabilities of death for depth factor
	pdep.33<-exp(b0+b2*mean(duration[]))
	pdep.32<-exp(b0+b1+b2*mean(duration[])+b3*mean(duration[]))
```

```
##Relative risks for marginal comparisons of duration and depth
RR.dur<-exp(b2+b3/2)
RR.dep<-exp(b1+b3/2)

##Relative risks for 3 hypothermia groups compared to standard
#cooling
RR1<-exp(b1)
RR2<-exp(b2)
RR3<-exp(b1+b2+b3)

##Risk differences for 3 hypothermia groups
RD1<-p1-p2
RD2<-p1-p3
RD3<-p1-p4

## Probability of any benefit for 3 hypothermia groups compared
#to standard
pRR1.1<-1-step(b1)
pRR2.1<-1-step(b2)
pRR3.1<-1-step(b1+b2+b3)

## Probability of RR < 0.9 for 3 hypothermia groups compared to
#standard
pRR1.90<-step(-0.1053605-b1)
pRR2.90<-step(-0.1053605-b2)
pRR3.90<-step(-0.1053605-(b1+b2+b3))

###Probability of increased mortality by 5% or more for 3
#hypothermia groups compared to standard

pRD1.INC05<-step(p2-p1-.05)
pRD2.INC05<-step(p3-p1-.05)
pRD3.INC05<-step(p4-p1-.05)

###Probability of decreased mortality by 1% or more for 3
#hypothermia groups compared to standard
pRD1.DEC01<-step(p1-p2-.01)
pRD2.DEC01<-step(p1-p3-.01)
pRD3.DEC01<-step(p1-p4-.01)

##Probabilities of any benefit and RR < 0.9 for marginal
#comparisons
pRR.dur.1<-1-step(b2+b3/2)
pRR.dur.90<-step(-0.1053605-(b2+b3/2))
```

```
    pRR.dep.1<-1-step(b1+b3/2)
    pRR.dep.90<-step(-0.1053605-(b1+b3/2))

    ##Probabilities of RD>0.01 and RD <(-0.05) for marginal
    #comparisons

    pRD.dur.INC05<-step(pdur.120-pdur.72-.05)
    pRD.dep.INC05<-step(pdep.32-pdep.33-.05)

    pRD.dur.DEC01<-step(pdur.72-pdur.120-.01)
    pRD.dep.DEC01<-step(pdep.33-pdep.32-.01)

}
```
